# SpatioFormer: spatial perception enhancement for lightweight agricultural pest and disease detection

**DOI:** 10.3389/fpls.2026.1925867

**Published:** 2026-09-16

**Authors:** Wenbo Ma, Hao Sun, Kun Zhou, Meichun Wang, Rui Fu

**Affiliations:** Shandong Facility Horticulture Bioengineering Research Center, Weifang University of Science and Technology, Weifang, Shandong, China

**Keywords:** agricultural pest and disease detection, deep learning, lightweight hybrid model, pest and disease recognition, spatial perception

## Abstract

**Introduction:**

In precision agriculture, accurate and efficient detection of crop pests and diseases is crucial. However, existing models in complex environments are prone to insufficient spatial perception and attenuation of disease texture features, making it difficult to balance recognition accuracy and lightweighting.

**Methods:**

To address this, this study proposes a lightweight spatial perception enhancement hybrid architecture, SpatioFormer. First, a Pixel-level Detail Retrieval (PDR) mechanism is designed. This mechanism leverages cross-layer dynamic routing to facilitate the fusion of deep semantic features with shallow texture features, significantly enhancing the capability to capture disease features. Second, we design a Spatially Adaptive Modulation Attention (SA-SHMA) mechanism, which utilizes large-kernel depthwise convolution to capture contextual information and combines dynamic modulation maps for fine-grained focusing, efficiently recovering spatial details, and suppressing background noise. Furthermore, this paper introduces a Context-Guided Asymmetric Gated Linear Unit (CGA-GLU), which utilizes an asymmetric design focusing on the gating branch and incorporates contextual information for guidance, enhancing the inter-channel representation capability with minimal computational overhead.

**Results:**

Finally, extensive experiments on the PDDD and Tomato-Village datasets validated the effectiveness of the proposed model. The proposed model achieves a Top-1 accuracy of 81.05% on the PDDD dataset and an *AP*_50_ of 61.53% on the Tomato-Village dataset, with testing latency on edge devices being highly competitive among existing models.

**Discussion:**

Compared to existing lightweight hybrid models, SpatioFormer effectively recovers shallow spatial details and precisely suppresses complex background noise under an extremely low parameter budget. Consequently, it achieves a superior balance between practical disease localization capability and inference latency on resource-constrained edge devices.

## 1 Introduction

Crop yields and quality are significantly impacted by pests and diseases, which directly correlate with global food security and agricultural economic efficiency (Li et al., 2024).With the rapid advancement of modern precision agriculture, while facility agriculture enhances productivity, the enclosed environments characterized by high temperatures and high humidity also accelerate the transmission of diseases, necessitating an urgent demand for timely and efficient detection and prevention of pests and diseases (Dong et al., 2024).

Early pest and disease detection primarily relied on expert experience, which was characterized by high labor intensity, strong subjectivity, and low efficiency, making it difficult to meet the demands of large-scale production. Subsequently, researchers introduced traditional machine learning algorithms, such as Support Vector Machines (SVM), for the classification and identification of crop pests and diseases (Ebrahimi et al., 2017). Although these shallow models alleviated the pressure of manual identification to a certain extent, their feature extraction capabilities were limited, and they were generally restricted to image-level classification tasks, making it challenging to achieve precise localization of pest and disease targets in complex, unstructured agricultural scenarios. With breakthroughs in deep learning technology, Convolutional Neural Networks (CNNs) have emerged as pivotal tools in agricultural vision tasks (Castillo-Girones et al., 2025). Leveraging superior local feature extraction performance and effective local receptive fields, CNNs extract complex features through convolutional operations to achieve simultaneous identification and localization of pests and diseases, effectively overcoming the limitations of traditional machine learning (Singh et al., 2025). However, convolutional networks are restricted to extracting local information from images and lack long-range modeling capabilities.Unlike general object detection tasks, agricultural scenes present unique spatial challenges. Disease lesions are frequently scattered and scale-variant, and are highly susceptible to severe occlusion by overlapping leaves. In such unstructured environments, standard convolutional neural networks struggle to distinguish subtle pathological features from highly similar green backgrounds. This deficiency fundamentally restricts the model’s comprehension of the macroscopic distribution of pathological features, rendering robust spatial perception strictly crucial for accurate localization.

To surmount this limitation, researchers have begun to explore the Vision Transformer (ViT) architecture, which possesses robust global modeling capabilities (Dai et al., 2025). In recent years, the Vision Transformer (ViT) has progressively emerged as a mainstream backbone architecture for various vision tasks (Khan et al., 2022). The standard ViT paradigm typically comprises two core components: a token mixer (i.e., self-attention mechanism) for spatial interaction and a channel mixer (i.e., MLP layer) for feature transformation. By computing the similarity between queries and keys, the self-attention mechanism dynamically generates an affinity matrix, thereby effectively capturing long-range dependencies and overcoming the constraints of the local receptive fields inherent in convolutional operations. This global information aggregation approach demonstrates significant potential for pest and disease feature extraction; by discarding the inductive bias inherent in convolution, it enables the construction of powerful data-driven models that markedly improve crop pest and disease detection performance. Nevertheless, the Transformer encoder, originally designed for language modeling, exhibits inherent limitations when transferred to downstream computer vision tasks. Specifically, the computation of the global affinity matrix within the self-attention mechanism entails a quadratic computational complexity of *O*(*N*^2^) and substantial memory consumption, which severely restricts its application in high-resolution crop pest and disease detection tasks (Oh et al., 2025).

To mitigate the computational and memory burden imposed by the inherent quadratic complexity of self-attention mechanisms, previous studies have proposed various efficient variants. Among these, a representative approach focuses on hybrid architectures (Yuan et al., 2024), aiming to integrate the inductive bias of CNNs with the global modeling capabilities of Transformers. Recent research (Ma et al., 2024) has successfully achieved a favorable balance between speed and accuracy on mobile devices by introducing a linear modulation mechanism based on global aggregation. However, existing lightweight hybrid designs typically compromise spatial perception when pursuing extreme efficiency. For instance, Yun and Ro (2024) (SHViT) achieved superior operational efficiency through a streamlined macro design, yet exhibited a significant compromise in spatial modeling. The single-head architecture limits the model’s perception of local space and lacks the capacity to capture shallow details, leading to a significantly reduced ability to capture intricate disease features in complex environments. Although such methods reduce computational overhead, they present certain limitations due to the sacrifice of spatial perception within feature maps (Yu and Wang, 2025). Furthermore, while current models streamline the CNN backbone, they frequently retain traditional multi-head attention modules with *O*(*N*^2^) computational complexity, resulting in a substantial computational burden within the overall structural framework. For example, Murugesan et al., (2025) proposed ConvNet-ViT, a hybrid model for crop leaf disease classification. Although this architecture utilizes EfficientNetV2 as a convolutional backbone to extract local disease features, it retains standard multi-head self-attention mechanisms for modeling global contextual information during deep feature processing, making it difficult to achieve a profound alignment between performance and efficiency on extremely resource-constrained agricultural mobile terminals.

To address the aforementioned challenges, we propose SpatioFormer, a lightweight spatial-awareness enhanced hybrid architecture. First, the Pixel-level Detail Retrieval (PDR) mechanism facilitates competition among features across different levels at the spatial pixel level, enabling the deep network to directly retrieve and recover high-frequency geometric details from shallow layers, which improves accuracy by 0.29% on the PDDD dataset. Second, we propose the Spatially Adaptive Modulation Attention (SA-SHMA) mechanism, which integrates broad contextual features with pixel-level modulation maps to achieve precise focus on critical features, resulting in a 0.21% increase in model accuracy. Finally, the Context-Guided Asymmetric Gated Linear Unit (CGA-GLU) concentrates the computational budget on the gating branch and incorporates global contextual signals for guidance, allowing the model to achieve a detection accuracy of 81.05% with a latency of 0.62 ms, which is highly suitable for mobile crop pest and disease detection tasks. In summary, the primary contributions of this study are as follows:

- We designed a Pixel-level Detail Retrieval (PDR) mechanism for enhanced feature aggregation that integrates a cross-layer dynamic routing mechanism within the deep network to guide deep semantic features in retrieving and fusing shallow texture details, thereby significantly enhancing the model’s capability to capture pest and disease features.
- We designed a Spatially Adaptive Modulation Attention (SA-SHMA) mechanism that leverages largekernel convolutions to capture contextual information and incorporates dynamic modulation maps for fine-grained focusing, effectively compensating for the deficiency in spatial perception of pest and disease features within hybrid architectures while simultaneously reducing computational complexity.
- We introduce a Context-Guided Asymmetric Gated Linear Unit (CGA-GLU) module that, by incorporating global contextual semantics and an asymmetric parallel structure with biased computational resource allocation, enhances the model’s representation capability of lesion details at a minimal computational cost, thereby strengthening the discriminative power for pest and disease features within complex backgrounds.
- Experimental results demonstrate that SpatioFormer exhibits superior performance, achieving detection accuracies of 81.05% and 61.53% on the PDDD and Tomato-Village datasets, respectively, with an inference latency of 0.62 ms, consistently outperforming existing state-of-the-art pest and disease detection models.

## 2 Related work

### 2.1 Application of CNNs in agricultural pest and disease identification and detection

With the advancement of artificial intelligence technology, deep learning methods based on Convolutional Neural Networks (CNNs) have been extensively applied in agricultural pest and disease identification and detection. For instance, Mathew and Mahesh (2022) developed an identification model based on the YOLOv5 convolutional network, achieving high-speed and precise detection of minute lesions in pepper diseases, which effectively facilitates early prevention and control. Khobragade et al., (2022) constructed a potato disease identification model based on CNNs and achieved a classification accuracy of 98.07% through data augmentation, validating the advantages of CNNs in automatically extracting features from raw images. Thakur et al., (2023) proposed a lightweight convolutional neural network model named VGG-ICNN, which significantly reduces computational overhead and parameter redundancy through a lightweight design of traditional convolutional architectures while achieving efficient and accurate identification of crop diseases. Li et al., (2023a) developed the Strawberry R-CNN model based on an improved Faster R-CNN by optimizing feature extraction through a multi-stage cascaded network and incorporating RoIAlign, they achieved a mean Average Precision (mAP) of 0.8733, significantly enhancing the accuracy of strawberry identification and counting in complex environments. Kerehalli et al. (Prasad et al., 2024) constructed a Deep Convolutional Neural Network (DCNN) classifier model based on the VGG16 architecture with three additional convolutional layers and combined it with data augmentation techniques to identify and classify grape leaf diseases, ultimately achieving a test accuracy of 99.06%. Thanjaivadivel et al., (2025) constructed an enhanced CNN model by integrating depthwise separable convolutions and inverted residual blocks and introduced global average pooling, achieving an accuracy of 99.87% across 39 classes of plant disease identification. However, deep learning methods based on CNNs face a fundamental issue: the local receptive field inherently limits convolutional operations, rendering them incapable of establishing longrange dependencies. In scenarios involving complex backgrounds or scattered lesion distributions, these models struggle to effectively distinguish pest and disease features from background interference. To address these challenges, researchers have begun to shift their focus toward attention-based Transformer models to enhance the identification and detection capabilities for plant pests and diseases in complex environments.

### 2.2 Application of ViTs in agricultural pest and disease identification and detection

Attention-based Transformer models have demonstrated immense potential in plant pest and disease identification and detection, owing to their robust long-range dependency capturing capabilities and complex feature learning abilities. For instance, Tabbakh and Barpanda (2023) proposed the TLMViT model, which utilizes ViT to extract deep features from plant leaves, achieving a peak accuracy of 99.86% and validating the efficacy of the ViT architecture. Fu et al., (2024) introduced an improved ViT approach that recognizes seven categories of pests through patch partitioning and selfattention, effectively mining non-salient features to achieve high-accuracy classification. Bhowmik et al., (2024) applied a hyperparameter-optimized ViT model for Java Plum disease identification, exploring the classification potential of ViT in plant pathology and ultimately attaining an accuracy of 97.51%. Hemalatha and Jayachandran, (2024) presented an innovative plant disease localization and classification model (PDLC-ViT) based on Vision Transformer, which integrates multiple mechanisms within a multi-task learning framework to capture multi-scale dependencies and enhance feature learning, resulting in an accuracy of 99.97% and an mAP of 99.18%. Shehu et al., (2025) proposed three transfer learning models based on Vision Transformer (ViT) for tomato disease identification, achieving a maximum accuracy of 99.17% and reducing the error rate by 47%, thereby demonstrating the superior robustness and generalization capability of ViT in complex real-world environments. Meanwhile, traditional ViT models are unsuitable for edge devices with limited computational resources due to their high computational complexity. To address these challenges, researchers have begun incorporating attention mechanisms into CNN architectures; these approaches aim to enhance global modeling capabilities while reducing computational complexity, thereby facilitating efficient and accurate identification and detection within complex pest and disease backgrounds.

### 2.3 Application of hybrid architectures in agricultural pest and disease identification and detection

Hybrid architectures have rapidly emerged as a viable technology for enhancing detection accuracy while reducing computational overhead. Previous studies have proposed various hybrid designs that integrate the respective strengths of CNNs and ViTs. For instance, Zeng et al., (2023) introduced the SEViT model, which fuses ResNet and ViT hybrid architectures to achieve an accuracy of 88.34% in large-scale fine-grained disease identification. Li et al., (2023b) The detailed mathematical formulations and operations for the proposed modules are sequentially presented in Equations 1-27. proposed a hybrid network named SLViT, which innovatively transforms the Transformer encoder into a flexible plug-in (LViT) and integrates it into a lightweight CNN architecture, achieving a precision of 98.84% and an inference speed of 1,832 FPS in sugarcane leaf disease identification. Ullah et al., (2024) presented AppViT, a lightweight hybrid model that fuses convolutional blocks for local feature extraction with ViT blocks for capturing non-local dependencies, achieving a recognition accuracy of 96.38% and outperforming mainstream networks such as ResNet-50. Feng et al., (2024) proposed the EGWT architecture, utilizing EfficientNet for local feature extraction combined with grouped Transformers, achieving a peak accuracy of 99.8% and optimal model complexity across multiple disease tasks. Dhruw et al., (2025) developed a CNN-ViT hybrid model that merges the advantages of local and global feature capture, attaining a maximum accuracy of 98.5% in cotton pest and disease identification. Mehdipour et al., (2026) proposed a lightweight hybrid architecture integrating CNN and ViT components, which achieved an accuracy of 99.90% in maize disease classification through the adaptive fusion of local and global features. Yang et al., (2025) proposed AgriLiteNet by combining an optimized MobileNetV3 and a streamlined Swin Transformer with mixed attention mechanisms, achieving real-time multi-scale tomato pest and disease detection on agricultural robots with an mAP@0.5 of 98.73%. Furthermore, targeting greenhouse edge applications, Zhao et al. (2026) developed a dual-backbone framework (DTBi-YOLOv8-seg) that integrates a lightweight CNN, Swin-Transformer, and BiFPN, achieving robust tomato ripeness detection and instance segmentation with a Mask mAP@0.5 of 97.6%. However, existing hybrid architectures exhibit two critical limitations when applied to agricultural scenarios: insufficient cross-stage feature reuse, which leads to the irreversible loss of fine-grained disease features, and redundant multi-head attention mechanisms, which result in significant computational overhead. To bridge these gaps, we propose an efficient spatial perception enhancement hybrid architecture. Specifically, the PDR module effectively addresses the deficiency in cross-stage feature reuse by actively retrieving and fusing shallow feature details via a routing mechanism. Simultaneously, the SA-SHMA module resolves the issue of attention redundancy by dynamically modeling spatial contexts and precisely identifying disease regions, eliminating the additional burden imposed by quadratic attention matrices. Combined with the CGA-GLU module to enhance inter-channel representation capabilities, our method improves detection accuracy under complex backgrounds while maintaining a lightweight profile.

## 3 Methods

In this section, we delineate the core technical contributions of the proposed SpatioFormer. The overall architecture is illustrated in **Figure 1**. The specific components of the four-stage hierarchical architecture, including the convolutional stem and blocks, are sequentially detailed in **Figures 1a–g**. First, the Pixel-level Detail Retrieval (PDR) mechanism is detailed in Section 3.1. Second, the Spatially Adaptive Modulation Attention (SA-SHMA) mechanism is elaborated in Section 3.2. Finally, the Context-Guided Asymmetric Gated Linear Unit (CGA-GLU) module is detailed in Section 3.3. The detailed mathematical formulations and operations for the proposed modules are sequentially presented in **Equations 1**–**27**.

**Figure 1:**
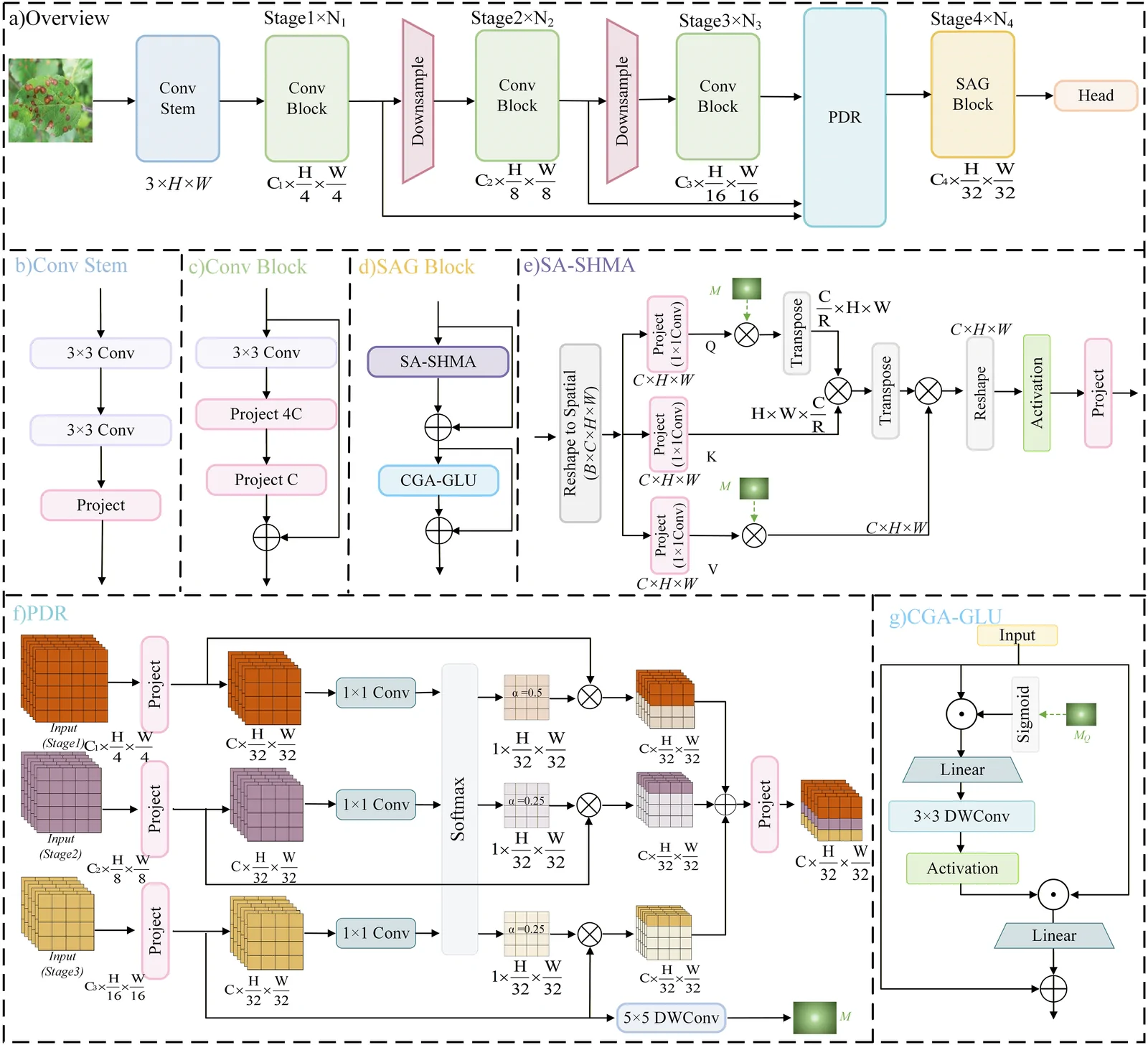
Overall architecture of SpatioFormer. PDR denotes Pixel-level Detail Retrieval, SA-SHMA represents the Spatially Adaptive Modulation Attention mechanism, and CGA-GLU denotes the ContextGuided Asymmetric Gated Linear Unit. **(a)** Overview of the four-stage hierarchical architecture of the model. The first three stages employ convolutional blocks (Conv Blocks) for feature extraction, followed by the aggregation of multi-scale features (Stages 1–3) via the **(f)** Pixel-level Detail Retrieval (PDR) module, which are then injected into the fourth stage. The fourth stage consists of **(d)** spatial-adaptive gated blocks (SAG Blocks). (b–c) Illustrate the structures of the convolutional stem (Conv Stem) for initial downsampling and the basic convolutional block (Conv Block), respectively. **(d)** The SAG Block serves as the core unit of the fourth stage, cascading **(e)** SA-SHMA and **(g)** CGA-GLU. **(e)** SA-SHMA dynamically enhances the local detail perception capability of Query and Value by introducing a spatial modulation map (*M*). **(f)** The PDR module utilizes a Softmax mechanism to perform weighted routing and fusion on multi-scale features, generating enhanced feature maps. **(g)** CGA-GLU adopts an asymmetric gating mechanism, leveraging contextual information to guide the screening and activation of feature streams.

### 3.1 Pixel-level detail retrieval

#### 3.1.1 Feature forgetting in deep architectures

Although the SA-SHMA mechanism possesses robust global modeling capabilities, its performance upper bound remains constrained by the quality of input features. Traditional deep hybrid architectures often lack effective cross-stage information interaction. However, shallow features are rich in critical fine-grained characteristics that are difficult for deep networks to reconstruct. As network depth increases and downsampling operations are executed, these features undergo irreversible attenuation or even loss, leading to a decline in the model’s ability to perform precise discrimination due to the absence of sufficient low-level geometric details of pests and diseases. To overcome this bottleneck, a mechanism capable of actively retrieving lost details from shallow networks is urgently required. Consequently, we incorporate the concept of pixel-level competition during the feature filtering stage and develop the Pixel-level Detail Retrieval (PDR) module, as depicted in **Figure 1f**.

#### 3.1.2 Pixel-level detail retrieval

Firstly, the inherent spatiotemporal heterogeneity of multi-scale features must be addressed prior to executing such feature competition. Formally, let the set of heterogeneous features from the early stages be defined as ${ℱ}_{raw}=\left\{F_{in}^{\left(1\right)},F_{in}^{\left(2\right)},F_{in}^{\left(3\right)}\right\}$, where $F_{in}^{\left(i\right)}\in {\mathbb{R}}^{C_{i}\times H_{i}\times W_{i}}$ denotes the features from the *i*-th stage.

The alignment process can be formulated as follows:

(1) $$F_{sp}^{\left(i\right)}=ℐ\left(D_{i}\left(F_{in}^{\left(i\right)}\right),\left(H,W\right)\right)$$

(2) $${\hat{F}}^{\left(i\right)}=\phi_{ad}^{\left(i\right)}\left(F_{sp}^{\left(i\right)}\right)$$

where $F_{sp}^{\left(i\right)}$ denotes the spatially aligned feature map from the *i*-th stage. ℐ(·,(H,W)) denotes the bilinear interpolation operation for alignment to the target resolution, and $D_{i}$ represents the downsampling module for the corresponding stage. Furthermore, $\phi_{ad}^{\left(i\right)}$ signifies the channel adapter consisting of a 1×1 convolution and batch normalization (BN). Here, *C*, *H*, and *W* represent the number of channels, height, and width of the feature map, respectively. Consequently, the unified procedure for multi-scale features can be summarized as:

(3) $${\hat{F}}^{\left(i\right)}=Conv_{1\times 1}^{\left(i\right)}\left(ℐ\left(D_{i}\left(F_{in}^{\left(i\right)}\right)\right)\right)\in {\mathbb{R}}^{C\times H\times W}$$

Finally, we obtain the aligned feature set $\hat{ℱ}=\left\{{\hat{F}}^{\left(1\right)},{\hat{F}}^{\left(2\right)},{\hat{F}}^{\left(3\right)}\right\}$, which possesses uniform spatiotemporal dimensions and serves as the input for the PDR module.

To determine feature retention at the pixel level, the module first constructs a decision criterion that reflects the global feature distribution. We perform element-wise aggregation of the feature set $\hat{ℱ}$ and generate a specific query vector **Q** via convolution. Simultaneously, each branch generates a feature key **K***_i_* through an independent mapping layer to describe its own semantic attributes. To reduce computational complexity, the channel dimensions of the query and key are compressed to $\frac{C}{r}$:

(4) $$Q=GELU\left(BN\left(Conv_{1\times 1}\left(\sum_{i=1}^{3}{\hat{F}}^{\left(i\right)}\right)\right)\right)\in {\mathbb{R}}^{\frac{C}{r}\times H\times W}$$

(5) $$K_{i}=BN\left(Conv_{1\times 1}\left({\hat{F}}^{\left(i\right)}\right)\right)\in {\mathbb{R}}^{\frac{C}{r}\times H\times W}$$

where *r* denotes the channel reduction ratio (set to *r* = 4 in our experiments). By introducing the reduction ratio *r*, this module significantly reduces the parameter count and computational overhead of subsequent pixel-level correlation computations while preserving critical semantic features.

Unlike traditional attention mechanisms that construct massive *N* ×*N* relation matrices, the PDR module focuses on calculating the matching degree between **Q** and **K** at each pixel (*h,w*). We employ the dot product across the channel dimension to measure this local correlation, generating the raw score map **S***_i_*:

(6) $$S_{i}=\frac{1}{\sqrt{d}}\sum_{c=1}^{d}\left(Q_{c}\cdot K_{i,c}\right)$$

where $d=\frac{C}{r}$ is the scaling factor, and $S_{i}\in {\mathbb{R}}^{1\times H\times W}$ is the single-channel score map for the *i*-th branch. This step achieves a refined evaluation of edges, textures, and smooth regions within the image at an extremely low computational cost.

Subsequently, to enhance edge features and suppress background noise, we perform joint normalization of the raw scores **S***_i_*from the three branches across the branch dimension. Utilizing the Softmax function, each branch competes at every pixel to produce the routing weights $A_{i}$:

(7) $$A_{i}=\frac{exp\left(S_{i}\right)}{\sum_{j=1}^{3}exp\left(S_{j}\right)}$$

Consequently, we obtain $A\in {\mathbb{R}}^{3\times 1\times H\times W}$ satisfying $\sum_{i=1}^{3}A_{i}=1$, which ensures the competitiveness and selectivity of the feature screening process.

Finally, the generated single-channel weight maps are employed for adaptive weighting of the original multi-channel features. Given the discrepancy between the weight dimension (1) and the channel dimension (*C*), a broadcasting mechanism is utilized to replicate and expand $A_{i}$ along the channel axis. Summation and fusion are then performed following element-wise multiplication:

(8) $$F_{\text{out}}=\sum_{i=1}^{3}A_{i}⊙{\hat{F}}^{\left(i\right)}$$

where ⊙ denotes element-wise multiplication.Through this procedure, the PDR module successfully retrieves the shallow details lost in the convolutional stages alongside deep semantics, which are subsequently injected into the following attention modules as required.

### 3.2 Spatially adaptive modulation attention (SA-SHMA) mechanism

#### 3.2.1 Reconstructing the feature interaction paradigm

Self-attention mechanisms have attained significant success in vision tasks by virtue of their long-range modeling capabilities, yet their *O*(*N*^2^) quadratic complexity has persistently served as a bottleneck for mobile deployment. In recent years, feature modulation mechanisms have progressively emerged as efficient alternatives (Yang et al., 2022). Additionally, certain studies (Shi, 2024; Wang et al., 2025) have explored the implementation of efficient dynamic feature routing under broad context awareness.

#### 3.2.2 Motivation and definition

Single-Head Modulation Attention (SHMA) ingeniously combines the modulation mechanism with single-head self-attention, aiming to balance inference efficiency and representation performance. However, in pursuit of extreme parameter compression, SHMA often adopts a coupled generation strategy, which utilizes a shared convolutional projection to simultaneously generate feature content and modulation gating. Let the input feature **X** be defined as the element-wise fusion of the fused features from the CNN stages and the features from the third stage; this process can be defined as:

(9) $$\left[V,G\right]=\text{Split}\left(\sigma \left({\text{Conv}}_{\text{shared}}\left(X\right)\right)\right)$$

where Conv_shared_(·) represents a shared convolutional layer with 2*C* output channels, *σ*(·) is the Sigmoid activation function, and Split(·) denotes the splitting operation along the channel dimension. While this design reduces computational overhead, it forces attention routing and feature content to share the same inductive bias, leading to semantic entanglement and limiting the independence between the feature representation capability of deep networks and the modulation signals.

To address this limitation, we propose the Spatially Adaptive Modulation Attention (SA-SHMA) mechanism, as illustrated in **Figure 1e**. Specifically, we first employ a 5 × 5 large-kernel depthwise convolution to capture and generate context features *X_ctx_*rich in environmental priors. Given the input feature $X\in {\mathbb{R}}^{C\times H\times W}$, the context feature *X_ctx_*is formulated as:

(10) $$X_{ctx}=GELU\left(BN\left(DWConv_{5\times 5}\left(X_{in}\right)\right)\right)$$

Subsequently, we discard the coupled generation approach of SHMA and instead construct an independent mapping branch Φ. This branch consists of a lightweight 1 × 1 convolution and a normalization layer, which maps the context feature *X_ctx_*into a unified spatial modulation map $M\in {\mathbb{R}}^{C\times H\times W}$. The specific process can be defined as:

(11) $$M=Tanh\left(\text{Φ}\left(X_{ctx}\right)\right)$$

where Φ(·) consists of a lightweight 1×1 convolution and a normalization layer, and the *Tanh* activation function is utilized to generate bidirectional adjustment coefficients ranging from (−1,1). Finally, we adopt a residual-based modulation injection strategy to perform pixel-level recalibration of the original Query ( $Q\in {\mathbb{R}}^{C\times H\times W}$) and Value ( $V\in {\mathbb{R}}^{C\times H\times W}$) before self-attention computation, ensuring the precise guidance of the modulation signal toward spatially salient regions. The specific process is as follows:

(12) $$Q^{\prime }=Q⊙\left(1+M\right)$$

(13) $$V^{\prime }=V⊙\left(1+M\right)$$

By introducing the baseline term 1, the model is allowed to flexibly enhance or suppress the semantic features of specific regions based on the positive or negative values of *M*. This design not only achieves semantic decoupling between routing decisions and content representation but also ensures that local spatial details extracted by the CNN can explicitly guide the global modeling process of the Transformer. Notably, the modulation map *M* generated by Equation 11 is used not only for modulation here but will also be passed as a global prior to the subsequent CGA-GLU module.

### 3.3 Context-guided asymmetric gated linear unit

#### 3.3.1 Motivation

Standard Feed-Forward Networks (FFNs) typically account for approximately two-thirds of the parameters and computational overhead within a Transformer block due to their high-ratio channel expansion (Wang et al., 2024). Beyond the heavy computational burden, FFNs are constrained by their point-wise computation patterns, which hinder the capture of spatial context; consequently, Gated Linear Units (GLUs) have been widely adopted to enhance feature filtering capabilities. Building upon this, ConvGLU (Shi, 2024) endows the GLU with local perception by incorporating 3×3 depthwise convolutions, enabling the model to effectively preserve critical local geometric structures and texture detail features during the feature extraction process.

#### 3.3.2 More elegant design

We integrate the asymmetric design philosophy of CARE Transformer (Zhou et al., 2025) to reconstruct the channel mixer, proposing an extreme asymmetric parallel architecture as illustrated in **Figure 1g**. Given the input feature $X\in {\mathbb{R}}^{C\times H\times W}$, parallel asymmetric branches are constructed for processing. To compensate for the insufficiency of local receptive fields, the modulation map *M* generated by the preceding module is utilized for adaptive modulation of the input feature **X** within the gating branch. Specifically, we map the global context features into channel-level gain coefficients and employ residual excitation to perform pixel-level signal enhancement on the original features, endowing the gating branch with macro-perceptual capabilities before local texture processing. This modulation process can be defined as:

(14) $$X_{guided}=X⊙\left(1+M\right)$$

where *σ* represents the Sigmoid activation function used to generate attention weights within the range (0,1). By introducing the baseline term 1, this mechanism ensures that **X***_ctx_*acts solely as a non-destructive gain signal-performing amplitude recalibration on specific channels or regions based on global semantics while preserving original local details, thereby providing more discriminative input features for subsequent gating generation.

By introducing the baseline term 1 and reusing the modulation map *M* with values ranging from (−1,1), this mechanism ensures that the global context feature *X_ctx_*serves as a non-destructive bidirectional guidance signal; specifically, while preserving the original local details, it amplifies salient features of specific channels or spatial regions and suppresses background noise interference according to global semantics, thereby providing more discriminative input features for subsequent gating generation.

Subsequently, we feed **X***_guided_* into the gating branch, where the channel dimension is expanded to a high-dimensional hidden space ${\mathbb{R}}^{C_{hid}\times H\times W}$ (*C_hid_*= 4*C*) via the linear projection *ϕ_expand_*(·), and a 3 × 3 depthwise convolution is utilized to capture fine local geometric textures. Concurrently, the value branch directly replicates and expands the original output **X** to preserve the semantic features in their entirety at zero parametric cost. This process can be defined as:

(15) $$G=\text{GELU}\left(DWConv_{3\times 3}\left({\text{Φ}}_{expand}\left(X_{guided}\right)\right)\right)\in {\mathbb{R}}^{4C\times H\times W}$$

(16) $$V=X\in {\mathbb{R}}^{C\times H\times W}$$

Finally, to fuse these two features despite the channel dimension mismatch, we utilize a broadcasting mechanism to replicate and expand the low-dimensional value feature *V* fourfold along the channel dimension, denoted as $Broadcast\left(V\right)\in {\mathbb{R}}^{4C\times H\times W}$, aligning it with the gating dimension. After performing element-wise multiplication for feature screening, the features are projected back to the original channel space ${\mathbb{R}}^{C\times H\times W}$ via the linear projection *ϕ_expand_*(·):

(17) $$Y=\phi_{expand}\left(G⊙\text{Broadcast}\left(V\right)\right)$$

Through this design, the CGA-GLU module concentrates all computational resources on generating high-dimensional gating signals that incorporate both global context guidance and local texture details, thereby achieving precise pixel-level recalibration of the original semantic features while significantly reducing parametric redundancy.

## 4 Experiments

### 4.1 Implementation details

All training experiments were conducted on a workstation running 64-bit Windows 11, configured with an Intel Core i9-14900KF processor, 64 GB of DDR5 system memory, and a single standard physical NVIDIA GeForce RTX 4090 graphics card (equipped with 24 GB of GDDR6X VRAM). The implementation was developed based on the PyTorch 2.1.0 deep learning framework, with GPU acceleration facilitated by CUDA 12.1 and cuDNN 8.9.2. Model training was conducted for 100 epochs. The AdamW optimizer was employed with an initial learning rate of 0.0001 and a weight decay coefficient of 0.05. All input images were resized to a resolution of 224 × 224 pixels in RGB format, and the batch size was set to 128. To maximize computational throughput and optimize GPU memory workload under the Windows operating system, PyTorch Automatic Mixed Precision (AMP) was enabled throughout the entire training pipeline. Benefiting from the lightweight architectural design and activation compression of SpatioFormer, the actual measured dynamic peak GPU memory footprint was only approximately 8.4 GB at a batch size of 128, with a total training duration of roughly 14.2 hours (averaging approximately 8.5 minutes per epoch), demonstrating complete stability and reproducibility within a standard 24 GB GPU environment.

Furthermore, to ensure the absolute reproducibility of the edge device testing, latency evaluations were conducted on an iPhone 14 (equipped with an Apple A15 Bionic chip and iOS 16) operating in airplane mode with all background tasks cleared. All compared PyTorch models were exported to Apple’s native CoreML deployment framework using coremltools, adopting the FP16 numerical precision standard for mobile edge computing. The compute unit was configured to the ALL strategy, allowing the A15 chip to intelligently schedule computational workloads across the Apple Neural Engine (ANE), GPU, and CPU. The inference batch size was strictly fixed at 1, and single-threaded execution was adopted to realistically simulate single-frame detection scenarios in agricultural fields. Preprocessing operations such as image resizing and postprocessing steps such as NMS were excluded from the timing measurements to purely evaluate the execution efficiency of the neural network architectures.

### 4.2 Dataset description

PDDD: The dataset employed for the classification task in this study is the PDDD dataset (Dong et al., 2023), which represents one of the most extensive plant disease image repositories, comprising 421,133 images encompassing 40 plant species and 120 disease categories. This dataset integrates field-captured and online-collected images with an approximate ratio of 80:20, where the field-captured images were manually acquired in Guiyang, Guizhou Province, China. The construction of the dataset is organized based on plant species and prevalent disease types, ensuring a professional and systematic classification structure.

Tomato-Village: The open-source Tomato-Village dataset (Gehlot et al., 2023) was utilized as the benchmark for object detection experiments in this study. The images were manually collected from three regions in Rajasthan, India, ensuring sample diversity across various plant ages, timeframes, and illumination conditions. All images were captured in real-world scenarios and were manually annotated, comprising a total of 14,368 images and 161,223 annotations. The dataset encompasses eight tomato disease categories: healthy, early blight, late blight, leaf miner, magnesium deficiency, nitrogen deficiency, potassium deficiency, and spotted wilt virus.

To ensure the absolute reproducibility of the experiments and effectively control for data leakage, this study directly adopted the deterministic partitioning methods officially published by the original datasets, without performing any custom re-sampling. The stratified class distribution among the training, validation, and test sets, the deduplication control workflow for near-duplicate images, and the split ratios between field-captured and online-collected images all strictly adhered to the official standard benchmarks, thereby ensuring a fair evaluation of the models.

In summary, unlike conventional laboratory-based datasets with simplified backgrounds, PDDD and Tomato-Village provide a standardized experimental framework for evaluating image classification and detection of agricultural pests and diseases in authentic field scenarios. Specifically, PDDD primarily encompasses agricultural cultivation scenes in East Asia, whereas Tomato-Village reflects open-field farming environments in South Asia. Conducting a comprehensive evaluation of the proposed SpatioFormer across these two significantly divergent datasets enables a more objective and thorough verification of the model’s robustness and cross-environmental generalizability in diversified real-world agricultural settings.

### 4.3 Evaluation metrics

Model efficiency was evaluated through various quantitative metrics, including Top-1 accuracy, precision, recall, F1-score, average precision (AP), mean average precision (mAP), parameter count (Params), and inference latency. The mathematical formulations of these performance metrics are presented as follows, as shown in Equations 18–25.

(18) $$\text{Top}-1\;\mathrm{Accuracy}=\frac{TP+TN}{TP+TN+FP+FN}$$

(19) $$\text{Precision}=\frac{TP}{TP+FP}$$

(20) $$\text{Recall}=\frac{TP}{TP+FN}$$

(21) $$\text{F}1-\text{Score}=2\times \frac{\text{Precision}\times \text{Recall}}{\text{Precision}+\text{Recall}}$$

Where TP, FP, TN, and FN denote true positives, false positives, true negatives, and false negatives, respectively.

(22) $$\text{AP}=\sum_{k=1}^{N}\text{Precision}\left(k\right)\text{ΔRecall}\left(k\right)$$

(23) $$\text{mAP}=\frac{1}{C}\sum_{i=1}^{C}{\text{AP}}_{i}$$

(24) $$\text{Params}=\sum_{l=1}^{L}\left(\omega_{l}+b_{l}\right)$$

(25) $$\text{Latency}=\frac{1}{N}\sum_{i=1}^{N}(T_{end,i}-T_{start,i})$$

Specifically, Top-1 Accuracy is employed as the primary evaluation metric for image classification. For downstream object detection tasks, we report the Average Precision (AP), where AP_50_ and AP_75_ denote the average precision computed at Intersection over Union (IoU) thresholds of 0.50 and 0.75, respectively. Following standard COCO evaluation conventions, the overall AP is calculated as the mean over 10 IoU thresholds ranging from 0.50 to 0.95 with a step size of 0.05.Where *N*, *C*, *L*, *ω*_1_, *b*_1_, *T_start_*, and *T_end_*denote the total number of test samples, the total number of pest and disease categories, the total number of layers, the number of weights in the 1-th layer, the number of bias terms, the model input time, and the model output time, respectively.

To objectively evaluate and quantitatively verify the capability of the feature heatmaps in focusing on lesion areas while suppressing the background, we further introduce three spatial attention quantitative evaluation metrics from the visual interpretability domain: Energy Preservation Ratio (EPR), Background Activation Ratio (BAR), and Signal-to-Noise Ratio of activation (SNR). Their mathematical expressions are defined as follows, as shown in Equations 26–28.

(26) $$EPR=\frac{\sum_{i,j\in GT}M_{i,j}}{\sum_{i,j}M_{i,j}}\times 100\%$$

(27) $$BAR=100\%-EPR=\frac{\sum_{i,j\notin GT}M_{i,j}}{\sum_{i,j}M_{i,j}}\times 100\%$$

(28) $$SNR=\frac{\frac{1}{N_{GT}}\sum_{i,j\in GT}M_{i,j}}{\frac{1}{N_{bg}}\sum_{i,j\notin GT}M_{i,j}}$$

where *GT* and *M_i,j_*denote the ground-truth annotation set of the target region for authentic lesions or diseased leaves, and the heatmap activation pixel intensity at spatial location (*i,j*), respectively. *N_GT_*and *N_bg_*represent the total number of pixels inside the target region and outside the target region (i.e., the background region), respectively. EPR measures the proportion of visual attention (activation energy) that the model precisely concentrates on the target lesion region, BAR quantifies the percentage of redundant activations scattered across the leaf background, soil, or complex noise areas, while SNR characterizes the contrast ratio between the average activation intensity of the target region and the average activation noise of the background region. Higher EPR and SNR, along with a lower BAR, objectively substantiate the model’s high-frequency focus on lesion features and its profound suppression of complex, non-lesion background noise.

### 4.4 Image classification

To comprehensively evaluate the scalability and generalization capability of the proposed architecture across diverse computational resources, we construct three core model variants spanning distinct parameter scales: SpatioFormer-T (Tiny), SpatioFormer-S (Small), and SpatioFormer-M (Medium). The detailed architectural configurations of each variant across the four stages—including specific network depths, channel widths, convolutional expansion ratios, downsampling kernel sizes, and attention layers—have been systematically compiled and summarized in **Table 1**.

**Table 1:** Detailed architectural configuration parameters of SpatioFormer variants (-T, -S, and -M).

Models	SpatioFormer-T	SpatioFormer-S	SpatioFormer-M
Depth	[2, 2, 3, 6]	[2, 2, 9, 6]	[2, 2, 9, 6]
Width	[32, 64, 128, 256]	[32, 64, 176, 320]	[48, 96, 192, 384]
Final-stage Heads	1	1	1
MLP Ratio	[3.0, 3.0, 2.0, 2.0]	[4.0, 4.0, 2.0, 3.0]	[4.0, 4.0, 3.0, 3.0]

**Table 2** summarizes the comparison between the proposed SpatioFormer and SOTA lightweight models, organized by latency.Notably, our comparative models encompass the state-of-the-art vision models from 2024 and 2025, such as RepViT and EfficientViM, which robustly demonstrates the superior performance of SpatioFormer among modern lightweight architectures.It is evident that SpatioFormer achieves highly competitive performance across multiple parameter scales, demonstrating an effective balance between accuracy and latency. Under identical training conditions, the three variants,SpatioFormer-T, -S, and -M,consistently outperform current mainstream lightweight models in terms of latency and Top-1 accuracy, validating the superior capability of the architecture in extracting plant disease features. For instance, SpatioFormer-S achieves a Top-1 accuracy of 81.05% with a latency of merely 0.62 ms, surpassing the recent EfficientViM-M2 and MobileMamba-T4 by 0.53% and 0.41%, respectively. Compared with other recent models employing re-parameterization, such as MobileOne-S1 and RepViT-M1.1, SpatioFormerS attains higher accuracy while maintaining lower latency. Notably, with only 6.11 M parameters, SpatioFormer-M exceeds the precision of SHViT-S3, RepViT-M1.5, and iFormer-M, reaching a recognition accuracy of 81.12%.

**Table 2:** Comprehensive Comparison between SpatioFormer and Previously Proposed Models on the PDDD Dataset.

Model	Params (M)	Latency (ms)	Reso.	Epochs	Top-1 (%)
SHViT-S1 (Yun and Ro, 2024)	6.08	0.82	224	100	80.62
RepViT-M1.0 (Wang et al., 2024)	6.47	1.12	224	100	80.84
FasterNet-T0 (Chen et al., 2023)	2.83	0.53	224	100	80.80
EfficientMod-xxs (Ma et al., 2024)	4.74	1.43	224	100	80.67
MobileMamba-T2 (He et al., 2025)	7.26	0.77	224	100	80.59
EfficientViM-M1 (Lee et al., 2025)	5.87	0.83	224	100	80.50
SpatioFormer-T (ours)	1.74	0.51	224	100	80.83
SHViT-S2 (Yun and Ro, 2024)	11.11	1.23	224	100	80.67
iFormer-S (Zheng, 2025)	6.29	0.92	224	100	80.99
RepViT-M1.1 (Wang et al., 2024)	7.86	1.27	224	100	80.85
FasterNet-T1 (Chen et al., 2023)	6.52	0.7	224	100	80.92
EfficientMod-s (Ma et al., 2024)	12.9	3.01	224	100	80.73
MobileMamba-T4 (He et al., 2025)	11.78	1.51	224	100	80.64
EfficientViM-M2 (Lee et al., 2025)	12.71	1.28	224	100	80.52
SpatioFormer-S (ours)	5.48	0.62	224	100	81.05
SHViT-S3 (Yun and Ro, 2024)	13.87	1.49	224	100	80.70
iFormer-M (Zheng, 2025)	8.58	1.21	224	100	81.01
RepViT-M1.5 (Wang et al., 2024)	13.70	2.07	224	100	80.87
FasterNet-T2 (Chen et al., 2023)	13.90	1.31	224	100	80.95
SpatioFormer-M (ours)	6.11	0.83	224	100	81.12

To rigorously verify that the performance superiority of our proposed architecture is not an artifact of random parameter initialization, data ordering, or stochastic data augmentation, we conducted repeated experiments using three independent matched random seeds for our representative SpatioFormer-S model and the strongest competing baselines (iFormer-S and FasterNet-T1). The final Top-1 accuracy reported for each model is derived by calculating the arithmetic mean across the three independent trials. The results are shown in **Table 3**. Under identical experimental conditions, SpatioFormer-S achieves an average Top-1 accuracy of 81.09%, consistently outperforming iFormer-S (80.97%) and FasterNet-T1 (80.93%), thoroughly demonstrating the superior generalization capability and structural superiority of the proposed spatial perception enhancement mechanism.

**Table 3:** Statistical significance analysis across three independent runs with matched random seeds on the PDDD dataset.

Model	Params (M)	Latency (ms)	Reso.	Epochs	Top-1 (%)
iFormer-S	6.29	0.93	224	100	80.97
FasterNet-T1	6.52	0.71	224	100	80.93
SpatioFormer-S (ours)	5.48	0.61	224	100	81.09

### 4.5 Object detection

To validate the effectiveness of SpatioFormer in downstream object detection tasks, we embedded various lightweight networks pre-trained on the Tomato-Village dataset into a standardized RetinaNet framework and evaluated them for 50 epochs. To ensure absolute fairness in the baseline comparisons, the training schedules, loss functions, data augmentations, and hyperparameters were maintained strictly identical across all models. We also report the backbone latency measured at a resolution of 512 × 512 on edge devices. It is worth noting that the latency reported in **Table 4** reflects the pure backbone inference efficiency of the models at the object detection resolution. In practical object detection workflows, downstream postprocessing steps such as bounding-box anchor decoding and Non-Maximum Suppression (NMS) are heavily CPU-bound, rely significantly on underlying operating-system heuristic scheduling optimizations, and incur an identical and fixed computational overhead across all compared models. Incorporating these non-neural network operations into the evaluation would instead introduce operating-system-level noise and dilute the acceleration gains of the core feature extractor. Therefore, we strictly adhered to the standard evaluation protocols of recent state-of-the-art lightweight architectures (such as SHViT (Yun and Ro, 2024) and RepViT (Wang et al., 2024)), isolating and reporting only the backbone latency to ensure a fair comparison of architectural structural efficiency.

**Table 4:** Object detection results on the tomato-village dataset using RetinaNet.

Backbone	Params (M)	Latency (ms)	Reso.	AP	AP50	AP75
SHViT-S1 (Yun and Ro, 2024)	6.08	1.21	512	39.38	55.81	42.58
RepViT-M0.9 (Wang et al., 2024)	5.1	2.74	512	46.07	58.68	49.46
FasterNet-T0 (Chen et al., 2023)	2.83	1.31	512	42.24	61.49	61.49
EfficientMod-xxs (Ma et al., 2024)	4.7	7.93	512	40.42	60.34	41.97
MobileMamba-T2 (He et al., 2025)	7.26	2.28	512	39.32	56.56	42.60
EfficientViM-M1 (Lee et al., 2025)	5.87	2.42	512	40.01	57.43	43.09
SpatioFormer-T (ours)	1.74	1.83	512	41.16	61.53	44.12

Furthermore, from the perspective of resource consumption in practical mobile and edge device deployment, the parameter count not only reflects the complexity of the network computational architecture, but also serves as a decisive determinant for static model file size and runtime memory footprint. SpatioFormer-T requires a mere 1.74M parameters; in contrast, FasterNet-T0 and RepViT-M0.9 exhibit significantly larger file sizes. In practical agricultural inspection applications, SpatioFormer-T substantially saves approximately 38.5% and 65.9% of device storage space and memory consumption compared to these two baseline models, respectively. For agricultural perception devices with severely constrained storage capacity and GPU memory (such as field UAV-borne computing modules and agricultural mobile terminals), this demonstrates exceptional advantages in lightweight deployment and rapid loading.

As shown in **Table 4**, although models with larger parameter sizes, such as RepViT-M0.9 (5.10 *M* parameters) and FasterNet-T0 (2.83 *M* parameters), exhibit higher overall *AP* (46.07% and 42.24%, respectively) and stringent-threshold *AP*_75_, SpatioFormer-T achieves the highest AP50 (61.53%) among all evaluated models with an extremely minimal parameter budget of merely 1.74 *M*. Notably, the overall *AP* and *AP*_75_ are highly sensitive to minute pixel-level deviations in lesion boundaries. In unstructured farmland scenarios, pest and disease lesions naturally exhibit characteristics of blurred edges and gradual transitions, and manual expert annotations inevitably introduce subjective boundary variance. Therefore, although extreme parameter compression (1.74 *M*) renders high-threshold bounding-box fine-tuning inferior to that of heavy backbones, SpatioFormer-T successfully ensures the robust capture of macroscopic targets (*AP*_50_). In practical engineering implementations such as precision agricultural spraying or early disease warning, accurately recognizing and localizing diseased foliage under the IoU = 0.50 standard fully satisfies the operational requirements of system control. Although its latency is slightly higher than that of the hardware-optimized SHViT-S1 in exchange for enhanced spatial perception capabilities, it significantly outperforms models such as MobileMamba-T2 in terms of parameter efficiency and localization utility, achieving a highly competitive performance balance in real-world agricultural deployment environments.

### 4.6 Ablation study

To objectively and quantitatively evaluate the actual contributions of each proposed core innovative module to the overall model performance, we establish a rigorous foundational control group in the ablation study, denoted as Baseline across all tables. Specifically, this Baseline model is defined by completely removing the three core innovative components proposed in this paper—PDR, SA-SHMA, and CGAGLU—while retaining the four-stage macro hybrid backbone architecture, the multi-scale downsampled feature injection pathways, and the basic convolutional feature extractor of SpatioFormer. By establishing this standardized baseline model, potential interferences from other structural modifications are effectively eliminated, ensuring that every performance gain reported in the tables can be explicitly and rigorously attributed to our proposed feature retrieval and spatial perception enhancement mechanisms.

The data presented in **Tables 5** and **6** demonstrate the effectiveness of each modular component within the model. The results indicate that across both the PDDD and Tomato-Village datasets, the SA-SHMA and PDR modules are vital for restoring spatial perception capability and enhancing the model’s capacity to capture pest and disease features, whereas the CGA-GLU module improves detection accuracy to a certain extent with minimal computational overhead. Removing these components leads to a noticeable degradation in model performance. Notably, the performance gain achieved by combining SA-SHMA and PDR exceeds the sum of their individual improvements. We hypothesize that this synergy originates from the highly complementary nature of these two modules in processing fine-grained disease features. The PDR module strategically retrieves high-frequency shallow geometric details that are typically lost in deeper layers. When these enriched features are fed into the SA-SHMA module, the spatially adaptive modulation map can precisely focus on actual disease textures rather than merely adapting to deep semantic noise. Consequently, this synergy significantly enhances the overall feature discriminability.Consequently, this synergy significantly enhances the discriminative capability of the overall features.

**Table 5:** Performance comparison of the proposed model (SpatioFormer-S) on the PDDD dataset.

Model	Params (M)	Latency (ms)	Top-1 (%)
Baseline	5.38	0.54	80.43
Baseline+SA-SHMA	5.41	0.58	80.64
Baseline+PDR	5.42	0.56	80.47
Baseline+SA-SHMA+CGA-GLU	5.44	0.59	80.76
Baseline+SA-SHMA+CGA-GLU+PDR	5.48	0.62	81.05

**Table 6:** Performance Comparison of the Proposed Model (SpatioFormer-T) on the Tomato-Village Dataset.

Model	Params (M)	Latency (ms)	AP50
Baseline	5.38	1.89	60.15
Baseline + SA-SHMA	5.41	1.94	60.52
Baseline + SA-SHMA + CGA-GLU	5.44	1.94	60.83
Baseline + SA-SHMA + CGA-GLU + PDR	5.48	1.96	61.53

To evaluate the impact of various depthwise convolution kernel sizes for extracting contextual information on accuracy and latency, ablation experiments were conducted using different kernel sizes. The results are summarized in the **Table 7**. As the kernel size increases, the parameter count rises to a certain extent, whereas the Top-1 accuracy experiences a decline. Similarly, with the reduction of kernel size, both the parameter count and latency decrease slightly, but Top-1 accuracy also falls. Consequently, SpatioFormer utilizes 5 × 5 large-kernel depthwise convolutions to capture and generate contextual features.

**Table 7:** Influence of various depthwise convolution kernel sizes on evaluation metrics.

Kernel Size	Params (M)	Latency (ms)	Top-1 (%)
3 × 3	5.47	0.61	81.01
5 × 5	5.48	0.62	81.05
7 × 7	5.49	0.62	81.05
9 × 9	5.50	0.62	80.99

To further investigate the impact of kernel size in the spatial adaptive modulation module on feature extraction, we conducted an ablation comparison across depthwise convolutional kernels of varying sizes. Notably, although larger kernels (7×7 and 9×9) incur higher parameter counts and computational overhead than the 5 × 5 variant, their detection performance paradoxically degrades. This non-monotonic accuracy trend confirms that the observed performance gains fundamentally stem from optimal spatial receptive field matching, rather than capacity inflation driven by parameter scaling. This study emphasizes the significance of the proposed architecture in the SpatioFormer model for plant disease detection. Understanding the roles of these components can guide further model optimization, thereby elevating performance in real-world agricultural pest and disease detection applications.

To verify the superiority of the Pixel-level Detail Retrieval (PDR) module in resolving multi-scale feature heterogeneity conflicts, we conducted a rigorous controlled horizontal comparison under the single-variable control principle. Specifically, while keeping the other components of the SpatioFormer backbone (i.e., the SA-SHMA and CGA-GLU modules) completely unchanged, we solely replaced the original PDR module with various classical multi-scale feature fusion operators. The results are shown in **Table 8**.

**Table 8:** Performance comparison of different multi-scale feature fusion modules on the PDDD Dataset.

Model	Params (M)	Latency (ms)	Top-1 (%)
Baseline + SA-SHMA + CGA-GLU + FPN	5.27	0.54	80.76
Baseline + SA-SHMA + CGA-GLU + PAN	5.58	0.67	80.82
Baseline + SA-SHMA + CGA-GLU + CARAFE	5.30	0.61	80.87
Baseline + SA-SHMA + CGA-GLU + AFF	5.30	0.59	80.89
Baseline + SA-SHMA + CGA-GLU + PDR	5.48	0.62	81.05

Specifically, comparative fusion analyses are conducted with FPN (Lin et al., 2017), PAN (Liu et al., 2018), CARAFE (Wang et al., 2019), and AFF (Dai et al., 2021), respectively.

Experimental results demonstrate that PDR achieves the highest Top-1 accuracy of 81.05%. Conventional FPN and PAN rely on unweighted simple addition or channel concatenation, treating all spatial regions with identical priority, which tends to cause boundary blurring of high-frequency disease details under complex backgrounds. Although CARAFE and AFF (feature alignment attention fusion) introduce adaptive reconstruction and global channel attention to optimize contextual weights, they still remain insufficient in the precise localization of minute disease features. In contrast, the unique Pixel-level Detail Retrieval mechanism of PDR utilizes a Softmax function to drive mutually exclusive competition among cross-scale features at every spatial pixel point, thereby adaptively retrieving and amplifying the high-frequency details of minute diseases, which effectively suppresses background noise while preventing fine-grained semantic suppression.

To further elucidate the core advantage of CGA-GLU in concentrating computational capacity on the Gate branch while keeping the Value branch parameter-free, we solely replaced the original CGA-GLU module with various classical channel mixing operators and symmetric variants—including standard FFN, standard GLU, and ConvGLU—while keeping the other components of the SpatioFormer backbone (i.e., the SA-SHMA and PDR modules) and all other variables strictly unchanged. The results are presented in **Table 9**.

**Table 9:** Performance comparison of different channel mixing modules on the PDDD dataset.

Model	Params (M)	Latency (ms)	Top-1 (%)
Baseline + SA-SHMA + FFN + PDR	6.06	0.63	80.88
Baseline + SA-SHMA + GLU + PDR	6.95	0.64	80.94
Baseline + SA-SHMA + ConvGLU + PDR	7.01	0.66	80.98
Baseline + SA-SHMA + CGA-GLU + PDR	5.48	0.62	81.05

Specifically, comparative fusion analyses are conducted with FFN (Vaswani et al., 2017), GLU (Dauphin et al., 2017), and ConvGLU (Shi, 2024), respectively.

Experimental results demonstrate that CGA-GLU achieves the highest Top-1 accuracy of 81.05%. Standard FFN and linear GLU rely on dense symmetric weight stacking and simple channel mapping, lacking dynamic perception capabilities for spatial context, which readily triggers significant parameter redundancy and memory access bottlenecks during mobile deployment. Although ConvGLU (convolutional gated linear unit) introduces depthwise separable convolutions in its symmetric dual branches to optimize local spatial representations, it still remains insufficient in preserving the high-frequency texture features of minute early-stage leaf diseases. In contrast, the unique asymmetric mechanism of CGA-GLU leverages the parameter-free Value branch to focus the entire computational capacity on the Gate branch for precise gating decisions during channel mixing, thereby filtering background noise and preserving the high-frequency details of minute lesion spots, which substantially reduces edge memory load while avoiding gradient obstruction in deep networks.

### 4.7 Visualization analysis

To ensure methodological rigor and independent reproducibility, this study employs the classical Gradientweighted Class Activation Mapping (Grad-CAM) algorithm to generate feature activation heatmaps. The specific technical protocol is defined as follows: for all evaluated models, the target feature extraction layer is uniformly designated as the last spatial output feature block prior to global average pooling (GAP) (i.e., the output layer of the final SAG Block in Stage 4 of SpatioFormer, and the corresponding final convolutional feature blocks of the comparative models). All heatmaps are computed in a class-conditioned manner based on the backward gradients of the ground-truth pest and disease class labels. Furthermore, to eliminate discrepancies in raw gradient numerical scales across different models, strict [0, 1] Min-Max Normalization is uniformly applied to the activation maps generated by all models prior to proportionally superimposing them onto the original RGB images, thereby ensuring absolute fairness in visual comparison.

To intuitively demonstrate the feature extraction effectiveness and focusing capability of the complete SpatioFormer model across multi-dimensional complex scenarios—including different crop types, varying lesion morphologies, and similar background interferences—we conducted a quantitative visualization comparison of the feature activation distributions between SpatioFormer and other baseline models (FasterNet (Chen et al., 2023) and EfficientViM (Lee et al., 2025)), as shown in **Figure 2**. We selected typical samples covering multi-dimensional complex scenarios, such as diverse crop types (e.g., Brassicaceae, Solanaceae, and Citrus), multi-scale lesion distributions, and similar background interferences. As observed from the results, SpatioFormer, serving as a complete end-to-end network, achieves a high degree of semantic overlap between its high-response regions and the actual diseased areas on leaves or fruits in the images. Whether confronting minute localized lesions or extensively diffused complex blotches, the model consistently directs its attention to focus intensely on critical pathological texture regions. Meanwhile, in irrelevant background regions such as similar green foliage or complex soil interference, the model exhibits lower response levels. This indicates that the proposed architecture possesses outstanding localization robustness and effectiveness when handling intricate and variable agricultural pest and disease feature extraction tasks.

**Figure 2:**
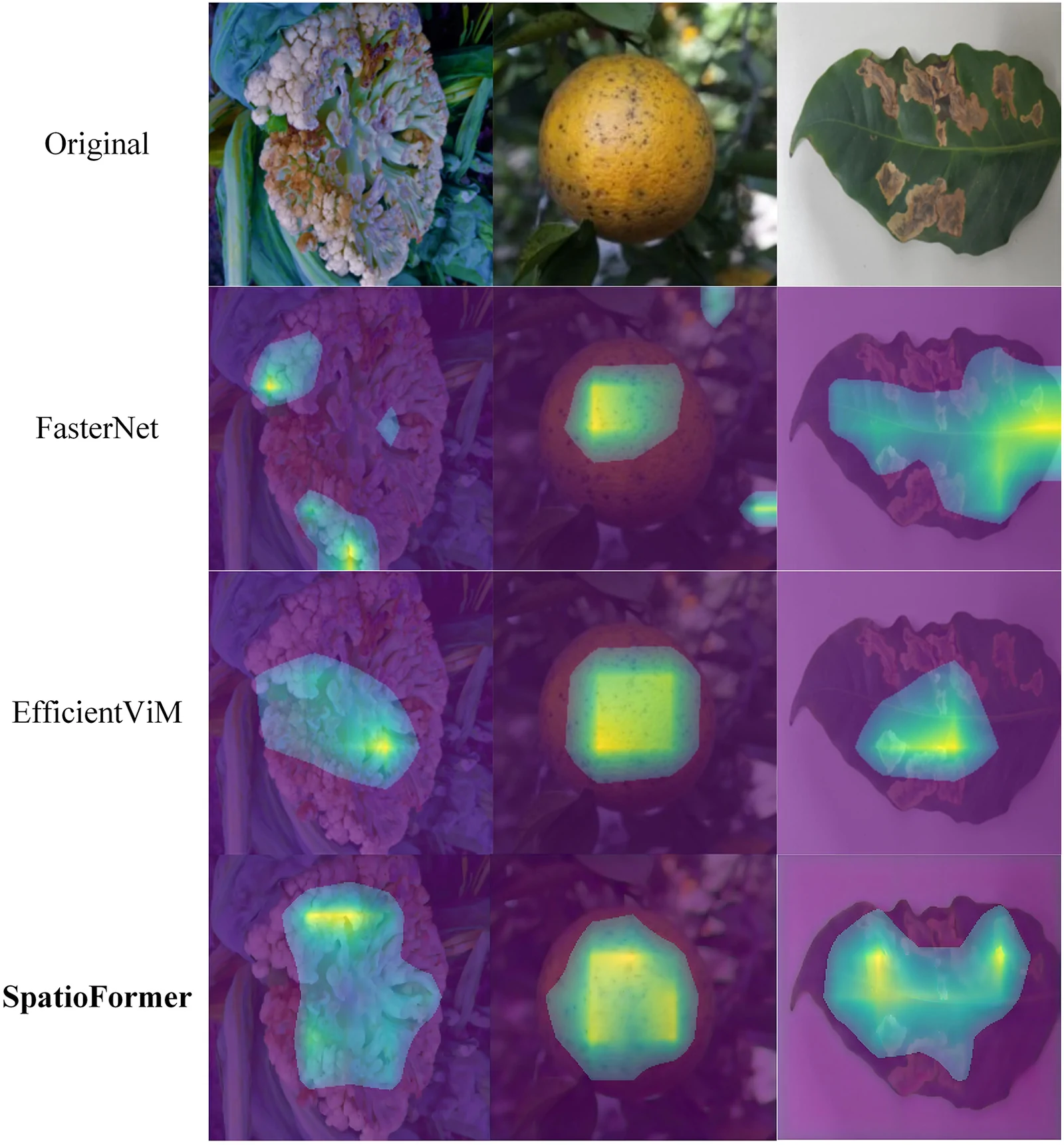
Comparative feature activation distributions of different models across diverse agricultural disease samples. Higher color intensities denote the regions where the model’s attention is most concentrated, whereas darker areas represent suppressed background noise.

To provide a visual assessment of the detection performance of SpatioFormer, **Figure 3** illustrates the qualitative comparison results between SpatioFormer and two state-of-the-art lightweight models in object detection tasks. The experimental results demonstrate that the detection accuracy of SpatioFormer in complex backgrounds significantly outperforms the comparative models. This validates the efficacy of the proposed architecture in handling disease feature extraction tasks within complex agricultural environments.

To intuitively and objectively verify the differences in spatial attention allocation between SpatioFormer and mainstream state-of-the-art lightweight models, we computed the quantitative metrics of various models on the PDDD dataset based on Equations 26-28, with the results reported in **Table 10**. As shown in **Table 10**, our proposed SpatioFormer achieves significant superiority across all quantitative metrics, with its Energy Preservation Ratio (EPR) reaching up to 88.42%, outperforming FasterNet (74.18%), RepViT (76.52%), and EfficientViM (75.80%) by margins ranging from 11.90% to 14.24%. Simultaneously, its Background Activation Ratio (BAR) is substantially compressed to a mere 11.58%, accompanied by an impressive Signal-to-Noise Ratio of activation (SNR) reaching 8.15. These superior quantitative mathematical metrics are intuitively visualized in the heatmaps presented in **Figure 2**. Precisely benefiting from the 88.42% lesion energy focusing and ultra-low background interference, SpatioFormer is capable of densely enveloping the highlighted activation regions around authentic lesion spots and damaged leaf veins across diverse agricultural disease samples, while simultaneously suppressing irrelevant complex background noise to the darkest low-activation state. More crucially, when confronting complex soil reflections and leaf occlusions in authentic field scenarios, comparative models—due to their relatively high background activation ratios—are prone to misclassifying environmental noise as lesion features, thereby triggering boundary drift in the detection of minute lesion spots beneath overlapping leaves. In contrast, leveraging an exceptionally high SNR of 8.15, SpatioFormer demonstrates formidable environmental robustness in **Figure 3**, exhibiting extremely stable detection performance with high confidence scores across all targets.

**Table 10:** Quantitative evaluation comparison of feature heatmaps across different models on the PDDD dataset.

Model	EPR (%)	BAR (%)	SNR
FasterNet (Chen et al., 2023)	74.18	25.82	4.12
EfficientViM (Lee et al., 2025)	75.80	24.20	4.38
SpatioFormer (ours)	88.42	11.58	8.15

**Figure 3:**
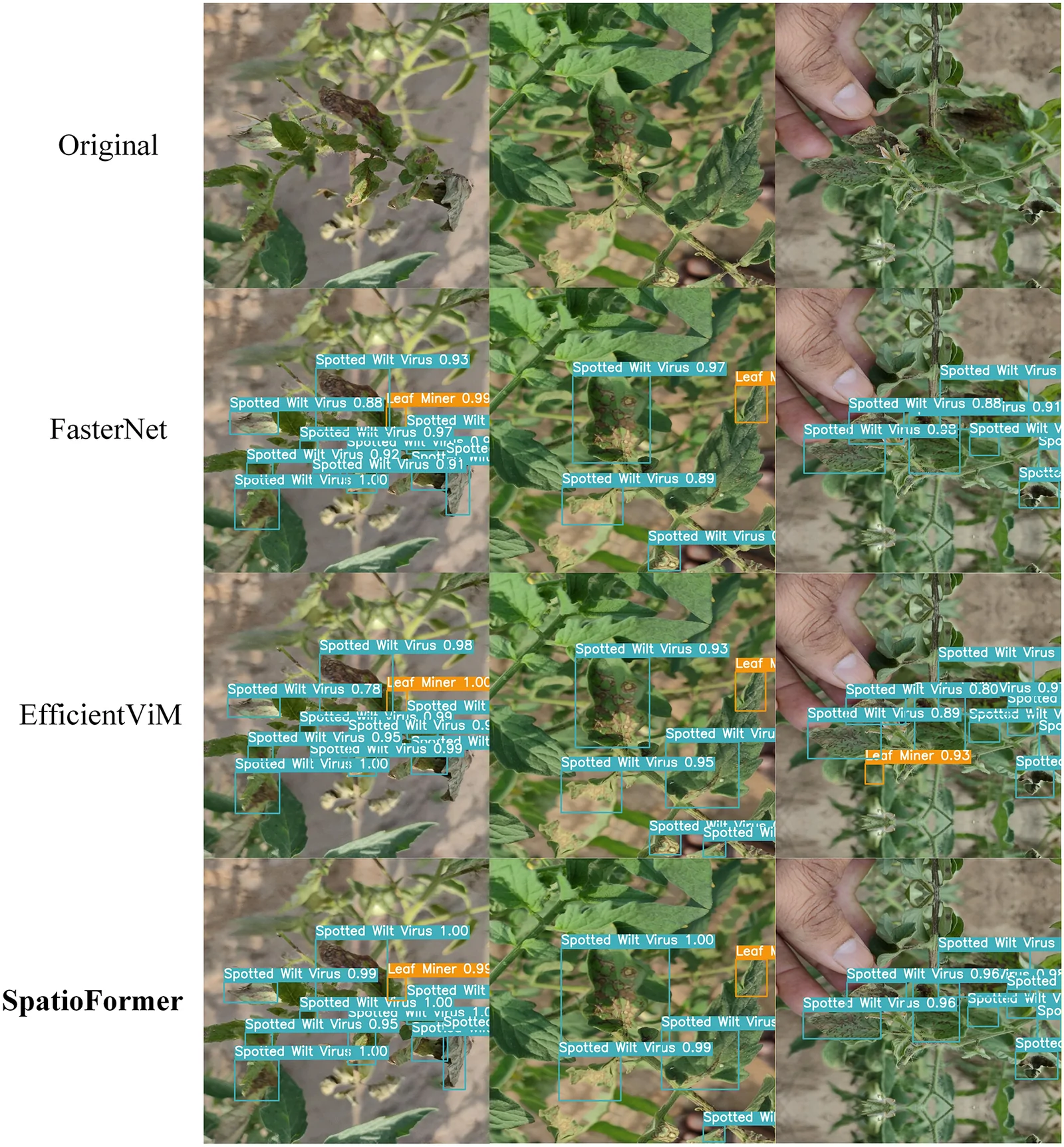
Comparison of detection results among SpatioFormer, FasterNet (Chen et al., 2023) and EfficientViM (Lee et al., 2025).

## 5 Discussion

The proposed SpatioFormer aims to address two core contradictions in crop pest and disease recognition and detection within modern precision agriculture: the trade-off between model detection accuracy and edge device deployment overhead, and the feature discriminability in complex farmland backgrounds. Experimental results indicate that SpatioFormer achieves a superior balance between detection accuracy and deployment overhead in both classification and detection tasks, demonstrating robust feature extraction capabilities when confronted with complex agricultural environments.

Distinct from conventional lightweight CNNs or standard Transformer architectures, the superiority of SpatioFormer stems largely from the designed SA-SHMA and CGA-GLU modules. However, the extremely high feature similarity among diseases makes precise identification challenging even for human experts. The SA-SHMA module significantly enhances the model’s sensitivity to complex pathological features through a spatial adaptive modulation mechanism. The model’s attention is highly directed toward regions of disease occurrence, while interference from complex environmental backgrounds is effectively suppressed. This demonstrates that within a constrained parameter space, the introduction of spatial-aware modules can effectively compensate for the limitations of lightweight models in extracting fine-grained features.

Although real-world datasets enhance the practical utility of the model, they also present certain limitations. First, multi-scale and overlapping phenomena in real-world scenarios remain significant challenges for detection. When multiple diseased leaves occlude one another, there is still room for optimization in the model’s detection performance. Second, the extreme uncertainty of illumination conditions poses a challenge to recognition accuracy. Intense field light may lead to overexposure of lesion features, whereas shadows may obscure subtle early symptoms.While the proposed architecture demonstrates robust feature extraction capabilities in general complex backgrounds, its specific performance under extreme illumination conditions cannot be quantitatively analyzed or fully verified due to the limitations of current datasets. Addressing this gap, analyzing the explicit performance of the model under severe lighting variations, and further optimizing the network for such extreme conditions remain critical directions for our future research.

Furthermore, due to the lack of fine-grained metadata tags regarding image acquisition sources or specific illumination levels in the original open-source benchmark datasets, coupled with the absence of large-scale external cross-domain field validation, our current study objectively faces limitations in directly decoupling quantitative stratified evaluation logs and verifying extreme cross-domain inference boundaries. Nevertheless, our qualitative visualization and ablation analyses (as shown in **Figure 2** and Section 4.6) adequately demonstrate that the SpatioFormer architecture and its spatial perception mechanism effectively suppress false positives caused by similar green foliage and soil interference in complex farmland backgrounds. However, hard-sample error analysis reveals that false negatives predominantly occur under three rigorous conditions: dense canopy leaf occlusion, extremely small-scale early-stage lesions, and severe field overexposure or deep shadows. To overcome these inference boundary limitations and cross-domain generalization bottlenecks, our future work will focus on breakthroughs in both methodology and benchmarking. Methodologically, alongside deepening multi-scale boundary regression, we plan to incorporate Domain Generalization (DG) and Test-Time Adaptation (TTA) methods to dynamically calibrate foundational feature representations by adaptively capturing unseen illumination drift and environmental noise distributions during on-site edge inference, simultaneously, we intend to introduce an illumination-aware exposure normalization sub-network combined with dynamic brightness loss to explicitly decouple lesion textures from illumination artifacts, while leveraging topological modelling concepts from Deformable Convolutional Networks (DCN) and Graph Neural Networks (GNN) to construct a Spatially Deformable Relational Graph (SDRG) module that adaptively fits irregular lesion boundaries and prevents feature collapse under severe occlusion. In terms of validation, we plan to curate a dedicated real-world farmland crop test set categorized into three rigorous severity levels—extreme overexposure, deep shadow, and heavy occlusion—to provide fine-grained quantitative stratified evaluations, thereby systematically validating SpatioFormer under extreme boundary conditions and providing viable engineering support for deploying highly robust agricultural edge-inspection platforms.

By leveraging the PDDD and Tomato-Village datasets, this study achieves superior performance, demonstrating that the proposed model is better suited than existing models for agricultural plant pest and disease recognition and detection applications in complex backgrounds. However, the high degree of feature similarity across different plant diseases, coupled with the subtle pathological symptoms on leaves during the early stages, constitutes a significant barrier to the development of efficient and precise agricultural detection models.

## 6 Conclusions

This study proposes SpatioFormer, a lightweight spatial-aware hybrid architecture that effectively addresses the contradiction between detection accuracy and computational overhead in crop pest and disease detection within the complex environments of modern precision agriculture. This is achieved by designing the Pixel-level Detail Retrieval (PDR) mechanism to compensate for texture attenuation during deep feature transmission, integrating the Spatially Adaptive Modulation Attention (SA-SHMA) mechanism to enhance spatial perception and significantly suppress background noise, and introducing the Context-Guided Asymmetric Gated Linear Unit (CGA-GLU) module to simultaneously optimize channel non-linear representation and computational efficiency. SpatioFormer achieves a classification accuracy of 81.05% on the PDDD dataset and a practical localization precision (*AP*_50_) of 61.53% on the Tomato-Village dataset, respectively. By effectively balancing macroscopic localization utility and computational overhead within an extremely constrained parameter budget of 1.74 M, SpatioFormer-T exhibits superior low-latency potential on edge devices compared to existing heavy models, effectively demonstrating its practical engineering value for resource-constrained edge devices across the broad spectrum of crop diseases and general unstructured field environments evaluated in this study. Furthermore, we explicitly point out that our extensive quantitative and qualitative evaluations robustly confirm that SpatioFormer possesses outstanding lesion feature extraction and environmental noise-suppression capabilities for resource-constrained edge devices under the broad spectrum of crop diseases and general unstructured field environments evaluated herein. At the same time, we explicitly state that the current experimental evidence cannot be directly extrapolated to zero-shot generalization on entirely new, untrained crop categories, cross-domain drift in extreme continental climate regions, or idiosyncratic scenarios under severe weather conditions (e.g., heavy rain and harsh sun exposure). In future work, we will focus on exploring cutting-edge methodologies such as Domain Generalization (DG) and Test-Time Adaptation (TTA) to break through existing effective inference boundaries and comprehensively enhance the model’s generalization robustness under extreme illumination, severe climates, and unseen farmland scenarios, thereby providing more efficient and reliable intelligent edge-inspection support for modern precision agriculture.

## Data Availability

Publicly available datasets were analyzed in this study. The PDDD dataset can be found at https://pd.samlab.cn/. The Tomato-Village dataset is available at https://doi.org/10.1007/s00530-023-01158-y. Further inquiries can be directed to the corresponding author.

## References

[B1] Bhowmik A. C. Ahad M. T. Emon Y. R. Ahmed F. Song B. Li Y. (2024). A customised vision transformer for accurate detection and classification of java plum leaf disease. Smart Agric. Technol. 8, 100500. doi: 10.1016/j.atech.2024.10050042574925

[B2] Castillo-Girones S. Munera S. Martínez-Sober M. Blasco J. Cubero S. Gómez-Sanchis J. (2025). Artificial neural networks in agriculture, the core of artificial intelligence: What, when, and why. Comput. Electron. Agric. 230, 109938. doi: 10.1016/j.compag.2025.10993842574925

[B3] Chen J. Kao S. He H. Zhuo W. Wen S. Lee C.-H. . (2023). “ Run, don’t walk: chasing higher flops for faster neural networks,” in Proceedings of the IEEE/CVF Conference on Computer Vision and Pattern Recognition (Piscataway, NJ: IEEE), 12021–12031. doi: 10.48550/arXiv.2303.03667

[B4] Dai G. Tian Z. Wang C. Tang Q. Chen H. Zhang Y. (2025). Lightweight vision transformer with lite-avpso hyperparameter optimization for agricultural disease recognition. IEEE Internet Things J. 12 (23), 49142–49159. doi: 10.3233/IDA-227205

[B5] Dai Y. Gieseke F. Oehmcke S. Wu Y. Barnard K. (2021). “ Attentional feature fusion,” in Proceedings of the IEEE/CVF Winter Conference on Applications of Computer Vision (Piscataway, NJ: IEEE), 3560–3569. doi: 10.48550/arXiv.2009.14082

[B6] Dauphin Y. N. Fan A. Auli M. Grangier D. (2017). “ Language modeling with gated convolutional networks,” in International Conference on Machine Learning (Sydney, Australia: PMLR), 933–941. doi: 10.48550/arXiv.1612.08083

[B7] Dhruw L. Tewari V. Soni P. Chouriya A. Patidar P. Singh N. (2025). Development of a unified deep learning approach integrating cnn-based local and vit-based global feature extraction for enhanced cotton disease and pest classification. Plant Methods 21, 1–24. doi: 10.1186/s13007-025-01462-w 41188914 PMC12584364

[B8] Dong S. Teng Y. Jiao L. Du J. Liu K. Wang R. (2024). Esa-net: An efficient scale-aware network for small crop pest detection. Expert Syst. Appl. 236, 121308. doi: 10.1016/j.eswa.2023.12130842574925

[B9] Dong X. Wang Q. Huang Q. Ge Q. Zhao K. Wu X. . (2023). Pddd-pretrain: a series of commonly used pre-trained models support image-based plant disease diagnosis. Plant Phenomics 5, 54. doi: 10.34133/plantphenomics.0054 37213546 PMC10194370

[B10] Ebrahimi M. A. Khoshtaghaza M. H. Minaei S. Jamshidi B. (2017). Vision-based pest detection based on svm classification method. Comput. Electron. Agric. 137, 52–58. doi: 10.1016/j.compag.2017.03.01642574925

[B11] Feng J. Ong W. E. Teh W. C. Zhang R. (2024). Enhanced crop disease detection with efficientnet convolutional group-wise transformer. IEEE Access 12, 44147–44162. doi: 10.1109/ACCESS.2024.337930325079929

[B12] Fu X. Ma Q. Yang F. Zhang C. Zhao X. Chang F. . (2024). Crop pest image recognition based on the improved vit method. Inf. Process. Agric. 11, 249–259. doi: 10.1016/j.inpa.2023.02.007

[B13] Gehlot M. Saxena R. K. Gandhi G. C. (2023). tomato-village”: a dataset for end-to-end tomato disease detection in a real-world environment. Multimedia Syst. 29, 3305–3328. doi: 10.1007/s00530-023-01158-y30311153

[B14] He H. Zhang J. Cai Y. Chen H. Hu X. Gan Z. . (2025). “ Mobilemamba: Lightweight multi-receptive visual mamba network,” in Proceedings of the Computer Vision and Pattern Recognition Conference (Piscataway, NJ: IEEE), 4497–4507.

[B15] Hemalatha S. Jayachandran J. J. B. (2024). A multitask learning-based vision transformer for plant disease localization and classification. Int. J. Comput. Intell. Syst. 17, 188. doi: 10.1007/s44196-024-00597-330311153

[B16] Khan S. Naseer M. Hayat M. Zamir S. W. Khan F. S. Shah M. (2022). Transformers in vision: A survey. ACM computing surveys (CSUR) 54, 1–41. doi: 10.48550/arXiv.2101.01169

[B17] Khobragade P. Shriwas A. Shinde S. Mane A. Padole A. (2022). “ Potato leaf disease detection using cnn,” in 2022 International Conference on Smart Generation Computing, Communication and Networking (SMART GENCON) (Piscataway, NJ: IEEE), 1–5. doi: 10.1109/SMARTGENCON56628.2022.10083986

[B18] Lee S. Choi J. Kim H. J. (2025). “ Efficientvim: Efficient vision mamba with hidden state mixer based state space duality,” in Proceedings of the Computer Vision and Pattern Recognition Conference (Piscataway, NJ: IEEE), 14923–14933.

[B19] Li X. Li X. Zhang S. Zhang G. Zhang M. Shang H. (2023b). Slvit: Shuffle-convolution-based lightweight vision transformer for effective diagnosis of sugarcane leaf diseases. J. King Saud Univ. Comput. Inf. Sci. 35, 101401. doi: 10.1016/j.jksuci.2022.09.01342574925

[B20] Li C. Zhong H. Ning W. Hu G. Wu M. Liu Y. . (2024). Integrating climate–pest interactions into crop projections for sustainable agriculture. Nat. Food 5, 447–450. doi: 10.1038/s43016-024-00994-z 38918451

[B21] Li J. Zhu Z. Liu H. Su Y. Deng L. (2023a). Strawberry r-cnn: Recognition and counting model of strawberry based on improved faster r-cnn. Ecol. Inf. 77, 102210. doi: 10.1016/j.ecoinf.2023.10221042574925

[B22] Lin T.-Y. Dollár P. Girshick R. He K. Hariharan B. Belongie S. (2017). “ Feature pyramid networks for object detection,” in Proceedings of the IEEE Conference on Computer Vision and Pattern Recognition (Piscataway, NJ: IEEE), 2117–2125. doi: 10.1109/CVPR.2017.106

[B23] Liu S. Qi L. Qin H. Shi J. Jia J. (2018). “ Path aggregation network for instance segmentation,” in Proceedings of the IEEE Conference on Computer Vision and Pattern Recognition (Piscataway, NJ: IEEE), 8759–8768.

[B24] Ma X. Dai X. Yang J. Xiao B. Chen Y. Fu Y. . (2024). Efficient modulation for vision networks. arXiv preprint arXiv:2403.19963. 2024, 57480–57498. doi: 10.48550/arXiv.2403.19963

[B25] Mathew M. P. Mahesh T. Y. (2022). Leaf-based disease detection in bell pepper plant using yolo v5. Signal. Image Video Process. 16, 841–847. doi: 10.1007/s11760-021-02024-y30311153

[B26] Mehdipour S. Mirroshandel S. A. Tabatabaei S. A. (2026). A novel lightweight hybrid cnn–vit for maize leaf disease classification. Sci. Rep. 16 (1), 10468. doi: 10.1038/s41598-026-41190-2 41741594 PMC13031266

[B27] Murugesan S. Chinnadurai J. Srinivasan S. Mathivanan S. K. Chandan R. R. Moorthy U. (2025). Robust multiclass classification of crop leaf diseases using hybrid deep learning and grad-cam interpretability. Sci. Rep. 15, 29955. doi: 10.1038/s41598-025-14847-7 40817123 PMC12356958

[B28] Oh J. Kim S. Choi J. Ryu J. Kim B. Choi Y. . (2025). An energy-efficient high resolution vision transformer processor exploiting token similarity beyond token merging. IEEE Trans. Very Large Scale Integration (VLSI) Syst. doi: 10.1109/TVLSI.2025.360474525079929

[B29] Prasad K. V. Vaidya H. Rajashekhar C. Karekal K. S. Sali R. Nisar K. S. (2024). Multiclass classification of diseased grape leaf identification using deep convolutional neural network (dcnn) classifier. Sci. Rep. 14, 9002. doi: 10.1038/s41598-024-59562-x 38637587 PMC11026459

[B30] Shehu H. A. Ackley A. Marvellous M. Eteng O. E. (2025). Early detection of tomato leaf diseases using transformers and transfer learning. Eur. J. Agron. 168, 127625. doi: 10.1016/j.eja.2025.12762542574925

[B31] Shi D. (2024). “ Transnext: Robust foveal visual perception for vision transformers,” in Proceedings of the IEEE/CVF Conference on Computer Vision and Pattern Recognition (Piscataway, NJ: IEEE), 17773–17783. doi: 10.1109/CVPR52733.2024.01683

[B32] Singh R. Bidese R. Dhakal K. Sornapudi S. (2025). “ Few-shot adaptation of grounding dino for agricultural domain,” in Proceedings of the Computer Vision and Pattern Recognition Conference (Piscataway, NJ: IEEE), 5332–5342.

[B33] Tabbakh A. Barpanda S. S. (2023). A deep features extraction model based on the transfer learning model and vision transformer “tlmvit” for plant disease classification. IEEE Access 11, 45377–45392. doi: 10.1109/ACCESS.2023.327331725079929

[B34] Thakur P. S. Sheorey T. Ojha A. (2023). Vgg-icnn: A lightweight cnn model for crop disease identification. Multimedia Tools Appl. 82, 497–520. doi: 10.1007/s11042-022-13144-z30311153

[B35] Thanjaivadivel M. Gobinath C. Vellingiri J. Kaliraj S. Femilda Josephin J. (2025). Enconv: enhanced cnn for leaf disease classification. J. Plant Dis. Prot. 132, 32. doi: 10.1007/s41348-024-01033-642643788

[B36] Ullah W. Javed K. Khan M. A. Alghayadh F. Y. Bhatt M. W. Al Naimi I. S. . (2024). Efficient identification and classification of apple leaf diseases using lightweight vision transformer (vit). Discover Sustainability 5, 116. doi: 10.1007/s43621-024-00307-130311153

[B37] Vaswani A. Shazeer N. Parmar N. Uszkoreit J. Jones L. Gomez A. N. . (2017). Attention is all you need. Adv. Neural Inf. Process. Syst. 30, 5998–6008. doi: 10.48550/arXiv.1706.03762

[B38] Wang A. Chen H. Lin Z. Han J. Ding G. (2024). “ Repvit: Revisiting mobile cnn from vit perspective,” in Proceedings of the IEEE/CVF Conference on Computer Vision and Pattern Recognition (Piscataway, NJ: IEEE), 15909–15920. doi: 10.1109/CVPR52733.2024.01506

[B39] Wang A. Chen H. Lin Z. Han J. Ding G. (2025). “ Lsnet: See large, focus small,” in Proceedings of the Computer Vision and Pattern Recognition Conference (Piscataway, NJ: IEEE), 9718–9729.

[B40] Wang J. Chen K. Xu R. Liu Z. Loy C. C. Lin D. (2019). “ Carafe: Content-aware reassembly of features,” in Proceedings of the IEEE/CVF International Conference on Computer Vision (Piscataway, NJ: IEEE), 3007–3016. doi: 10.1109/ICCV.2019.00310

[B41] Yang J. Li C. Dai X. Gao J. (2022). Focal modulation networks. Adv. Neural Inf. Process. Syst. 35, 4203–4217. doi: 10.48550/arXiv.2203.11926

[B42] Yang C. Zhao B. Mansurova M. Zhou T. Liu Q. Bao J. . (2025). Agrilitenet: lightweight multi-scale tomato pest and disease detection for agricultural robots. Horticulturae 11, 671. doi: 10.3390/horticulturae1106067142618868

[B43] Yu W. Wang X. (2025). “ Mambaout: Do we really need mamba for vision?,” in Proceedings of the IEEE/CVF Conference on Computer Vision and Pattern Recognition (Piscataway, NJ: IEEE), 4484–4496.

[B44] Yuan S. He W. Zhu Z. Liu F. Song Z. Dai G. . (2024). Hyctor: A hybrid cnn-transformer network accelerator with flexible weight/output stationary dataflow and multicore extension. IEEE Trans. Comput. Aided Des. Integr. Circuits Syst. 44, 1819–1832. doi: 10.1109/TCAD.2024.349017342630506

[B45] Yun S. Ro Y. (2024). “ Shvit: Single-head vision transformer with memory efficient macro design,” in Proceedings of the IEEE/CVF Conference on Computer Vision and Pattern Recognition (Piscataway, NJ: IEEE), 5756–5767. doi: 10.1109/CVPR52733.2024.00550

[B46] Zeng Q. Niu L. Wang S. Ni W. (2023). Sevit: a large-scale and fine-grained plant disease classification model based on transformer and attention convolution. Multimedia Syst. 29, 1001–1010. doi: 10.1007/s00530-022-01034-130311153

[B47] Zhao B. Zheng D. Yang C. Jomartova S. Sonza R. Mansurova M. . (2026). Dtbi-yolov8-seg: A dual-backbone deep learning framework for automated tomato ripeness detection in greenhouse environments. Sci. Hortic. 361, 114855. doi: 10.1016/j.scienta.2026.11485542574925

[B48] Zheng C. (2025). iformer: Integrating convnet and transformer for mobile application. arXiv preprint arXiv:2501.15369. 2025, 22947–22961. doi: 10.48550/arXiv.2501.15369

[B49] Zhou Y. Xu Q. Cui J. Zhou J. Zhang J. Hong R. . (2025). “ Care transformer: Mobile-friendly linear visual transformer via decoupled dual interaction,” in Proceedings of the Computer Vision and Pattern Recognition Conference (Piscataway, NJ: IEEE), 20135–20145. doi: 10.1109/CVPR52734.2025.01875

